# A Systematic Approach to Intraoperative Venous Congestion in the Deep Inferior Epigastric Artery Perforator (DIEAP) Flap

**DOI:** 10.7759/cureus.49100

**Published:** 2023-11-20

**Authors:** Tony João, Vera Eiró, Ruben Nogueira, João Tavares, Rui Bastos

**Affiliations:** 1 Plastic and Reconstructive Surgery, Centro Hospitalar Lisboa Ocidental, Lisbon, PRT

**Keywords:** venous congestion, dieap, reconstruction, breast, microsurgery

## Abstract

The deep Inferior epigastric artery perforator (DIEAP) flap is currently the gold standard for autologous breast reconstruction. This flap is susceptible to venous congestion, which can result in partial or complete flap loss. Apart from external causes, venous congestion may be caused by the flap's vascular architecture, either due to a dominance of the superficial venous system or due to impaired communication between the superficial and deep venous systems. This inefficient vascular architecture can be detected during surgery, and the venous outflow drainage can be improved through several techniques. We present two case reports of intraoperative venous congestion. In the first case, we performed an intra-flap rerouting, through a venous anastomosis between the superficial and the deep venous systems. In the second case, an extra-flap rerouting was executed, through a venous anastomosis between the superficial venous system and a recipient vein. We present the current institutional approach to DIEAP flap breast reconstruction, incorporating surgical insights for addressing intraoperative venous congestion.

## Introduction

The deep inferior epigastric artery perforator (DIEAP) flap was first described by Koshima and Soeda in 1989 [1]. In 1994, Allen and Treece demonstrated the applicability of the DIEAP flap in breast reconstruction [2], which is currently assumed as the gold standard for autologous breast reconstruction [3,4]. This technique significantly decreased the abdominal wall complication rate when compared to the transverse rectus abdominis myocutaneous (TRAM) flap [3,5]. However, there is an increased risk of venous congestion, this being the most frequent vascular complication of the DIEAP flap with partial or complete flap loss potential [6,7].

Whereas the arterial inflow of the infraumbilical abdominal wall is mainly granted by the deep vascular system through the deep inferior epigastric artery (DIEA), the venous outflow is mainly drained by the superficial vascular system through the superficial inferior epigastric vein (SIEV) [3,5,8-10]. The superficial and deep venous systems are connected through some communicating veins that allow free blood flow between each system [11,12]. During the dissection of the DIEAP flap, the SIEV is ligated, redirecting the venous blood through the communicating veins to the deep inferior epigastric venae (DIEV) comitantes, via the venae comitantes of the selected paraumbilical perforator [4,6,8,10,12-14]. In the end, the flap’s venous outflow is granted by two DIEV comitantes that communicate with each other and run in close relation with the DIEA [14].

The flap's vascular architecture can lead to intraoperative venous congestion in 2% to 8% of cases [8,14,15]. This condition may be the result of impaired communicating veins between the deep and superficial venous systems. In these circumstances, the problem can be solved by performing a shunt between the two systems (intra-flap rerouting). However, the venous congestion can be due to an augmented resistance of the DIEV comitantes to the venous outflow. In those cases, the DIEV will not be able to drain the flap, even after performing the intra-flap rerouting. An extra-flap rerouting is required, accomplished by anastomosing a recipient vessel directly to the superficial system [14]. We, hereby, report two cases of DIEAP flaps with intraoperative venous congestion. In the first case, the flap was saved by an intra-flap rerouting, and in the second one by an extra-flap rerouting.

## Case presentation

Case report 1

**Figure 1:**
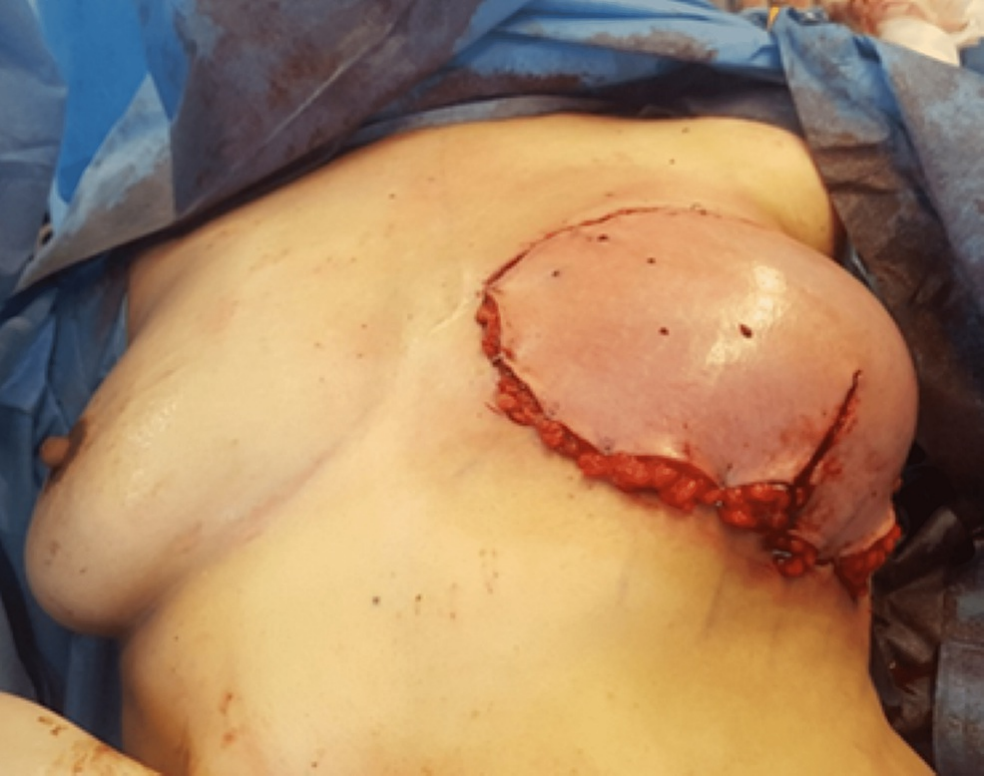
Intraoperative signs of congestion (case report 1).

**Figure 2:**
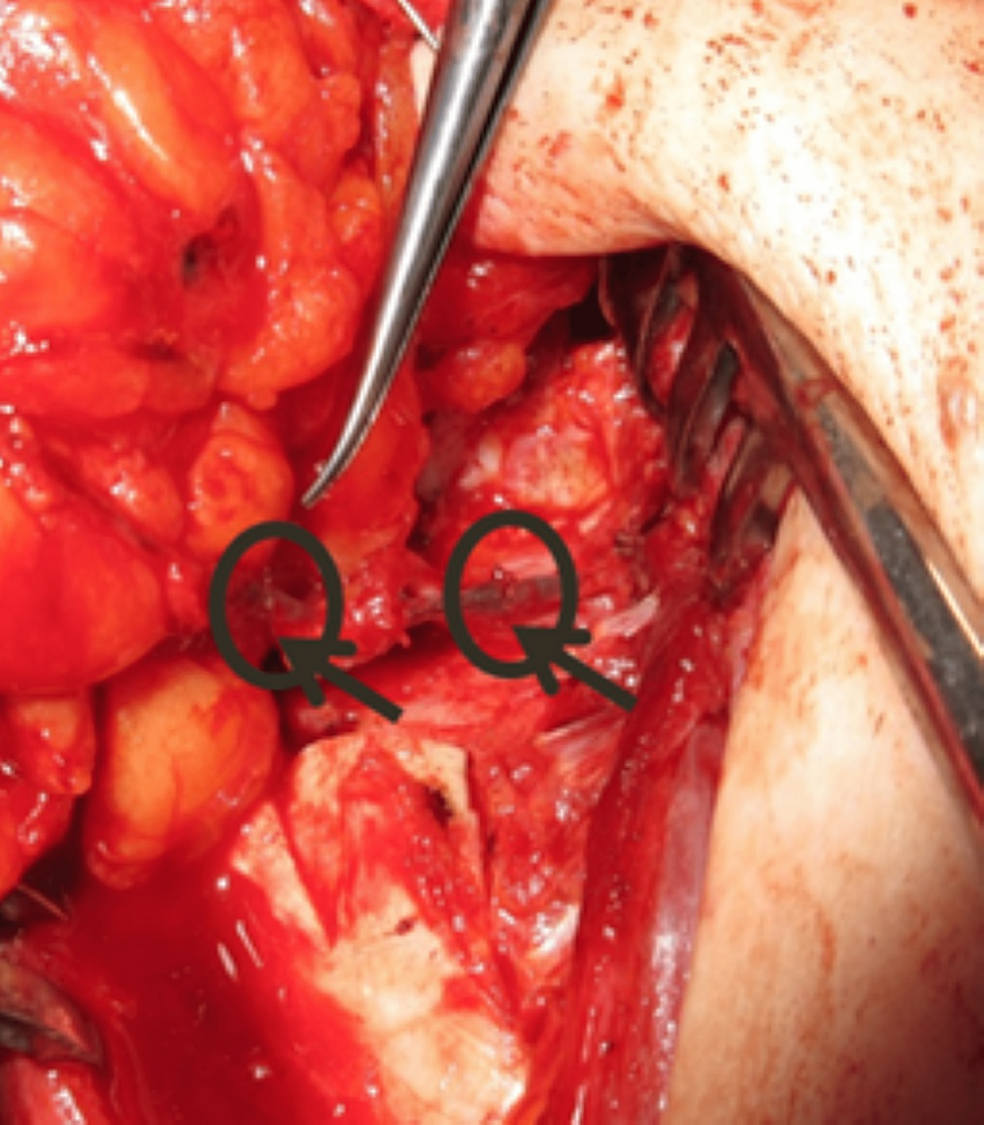
Reverse-flow venous anastomosis between the ipsilateral SIEV and the unused DIEV comitantes (case report 1). SIEV, superficial inferior epigastric vein; DIEV, deep inferior epigastric venae

**Figure 3:**
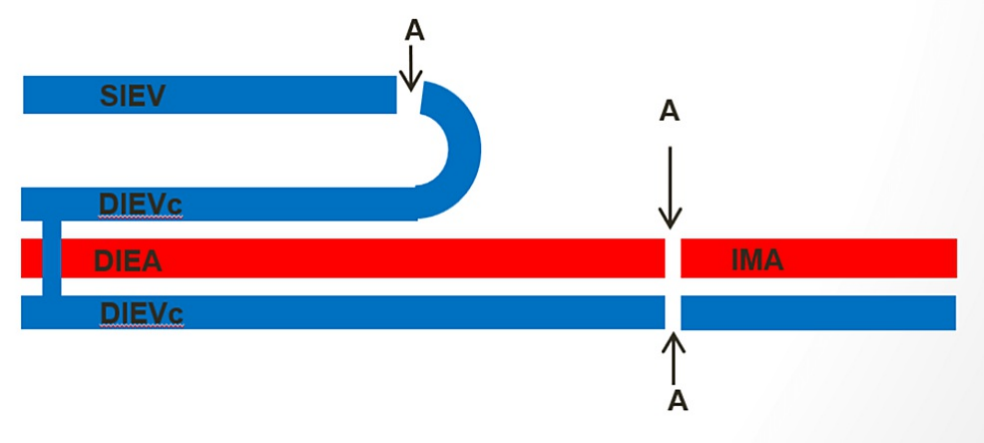
Scheme of venous anastomosis (case report 1). Image credit: Rui Bastos. A, anastomosis; DIEA, deep inferior epigastric artery; DIEVc, deep inferior epigastric venae comitantes; IMA, internal mammary artery; SIEV, superficial inferior epigastric vein

**Figure 4:**
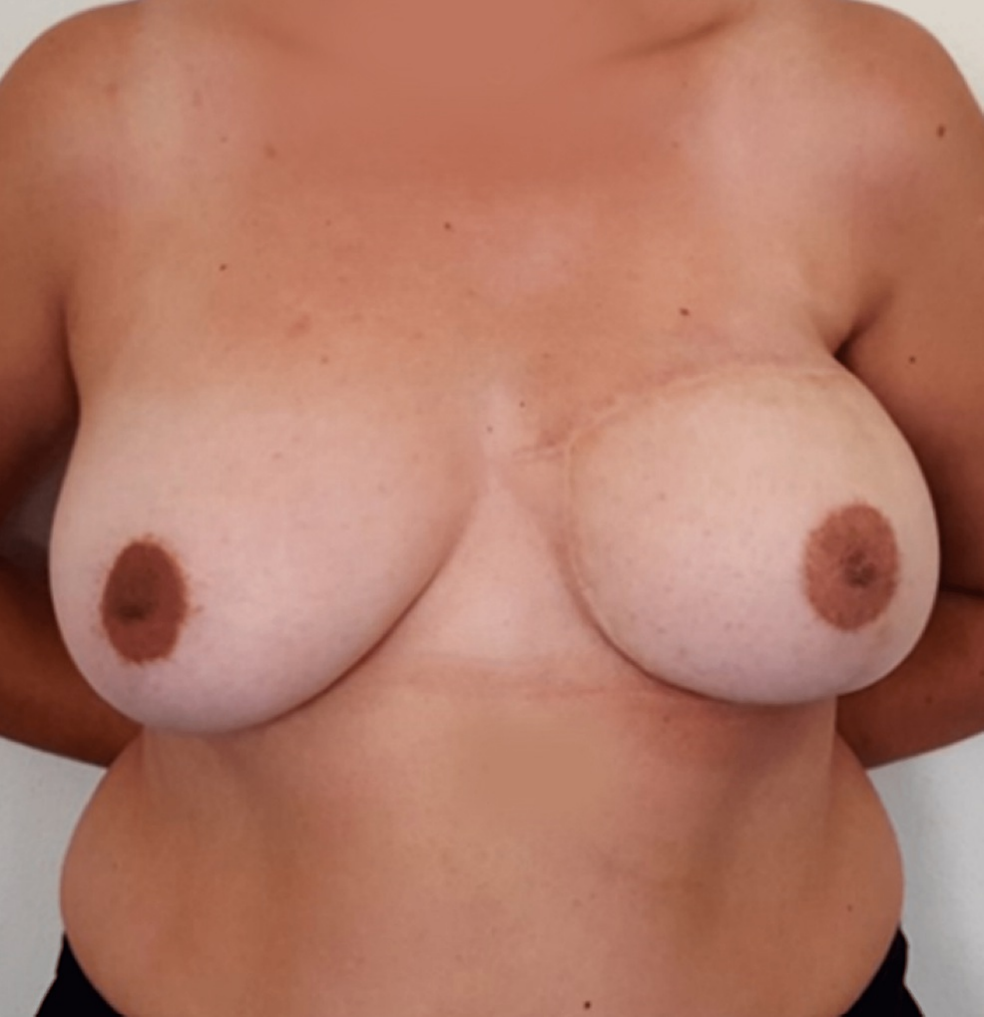
Postoperative result (case report 1).

A 39-year-old woman underwent delayed left breast reconstruction with a DIEAP flap after modified radical mastectomy, chemotherapy, and radiotherapy for invasive carcinoma. Preoperative computed tomographic angiography (angio-CT) was performed for surgical planning and perforator selection. The largest perforator with the best Doppler signal was found on the left medial row. During dissection, the SIEVs were preserved bilaterally. While one team was dissecting the flap, a second team was preparing the left internal mammary vessels in the recipient area. After completing the dissection of the flap, the pedicle was immediately ligated, and the flap was transposed to the recipient area. An arterial anastomosis was performed between the DIEA and the internal mammary artery (IMA), and a venous anastomosis was conducted between one of the DIEV comitantes and the internal mammary vein (IMV). After the anastomoses were completed and checked for appropriate patency, the signs of venous congestion started to become evident (Figure 1). As we suspected impaired communication between the superficial and deep venous systems, we decided to perform an intra-flap rerouting through a reverse-flow venous anastomosis between the ipsilateral SIEV and the unused DIEV comitantes (Figure 2). This venous anastomosis allowed a venous outflow rerouting from the superficial venous system to the deep system through one of the DIEV comitantes, which, in turn, communicates with the other DIEV comitantes already anastomosed to the IMV (Figure 3). There was an immediate recovery of the intraoperative venous congestion signs. A daily dose of 100 mg of acetylsalicylic acid and 40 mg of enoxaparin were administrated for six days. No sequelae were observed in the postoperative period (Figure 4).

Case report 2

**Figure 5:**
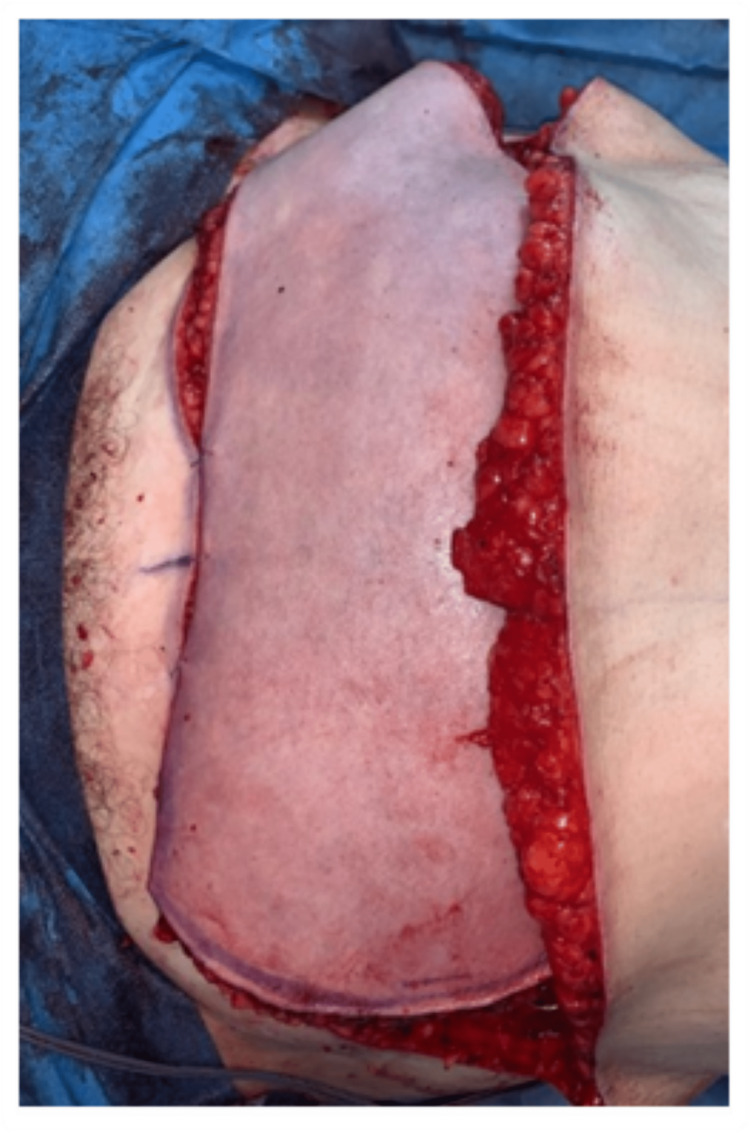
Intraoperative signs of congestion (case report 2).

**Figure 6:**
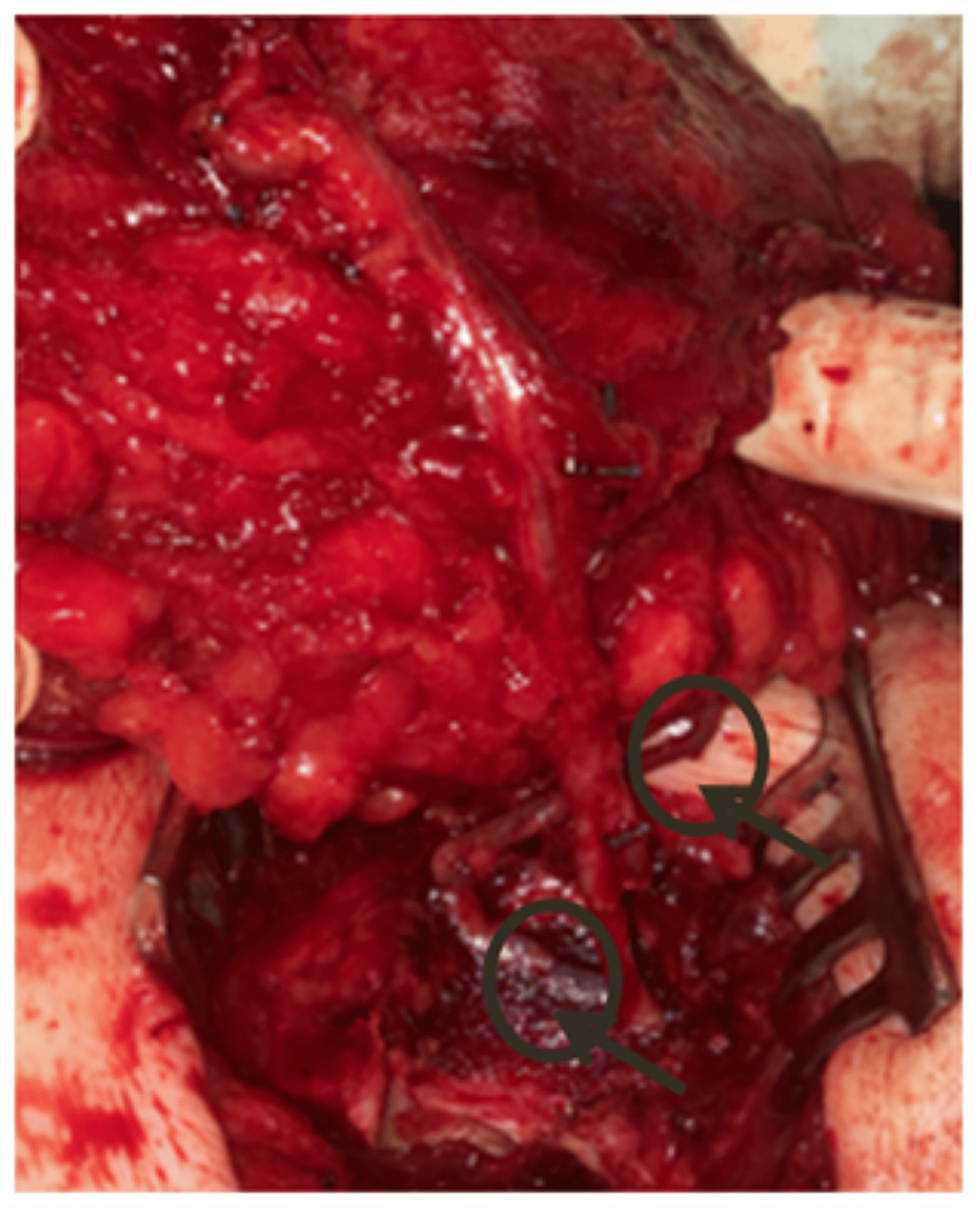
Venous anastomosis between the SIEV and the IMV (case report 2). IMA, internal mammary artery; SIEV, superficial inferior epigastric vein

**Figure 7:**
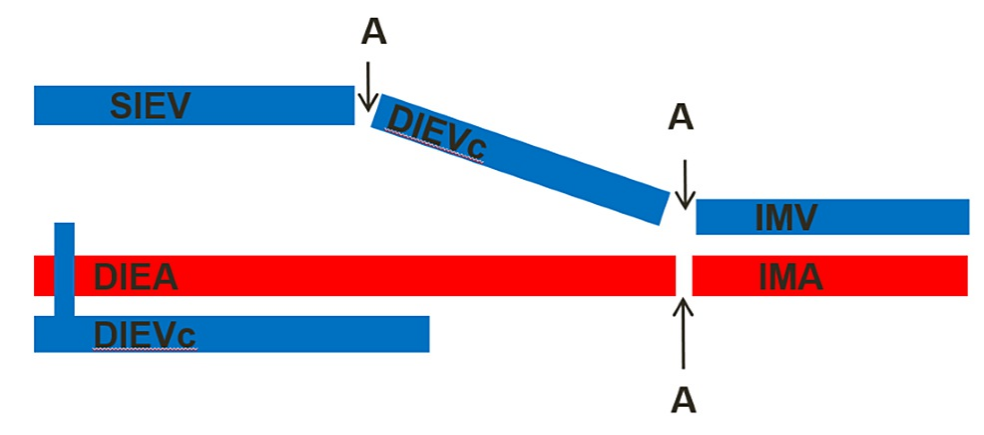
Scheme of venous anastomosis (case report 2). Image credit: Rui Bastos. A, anastomosis; DIEA, deep inferior epigastric artery; DIEVc, deep inferior epigastric venae comitantes; IMA, internal mammary artery; IMV, internal mammary vein; SIEV, superficial inferior epigastric vein

**Figure 8:**
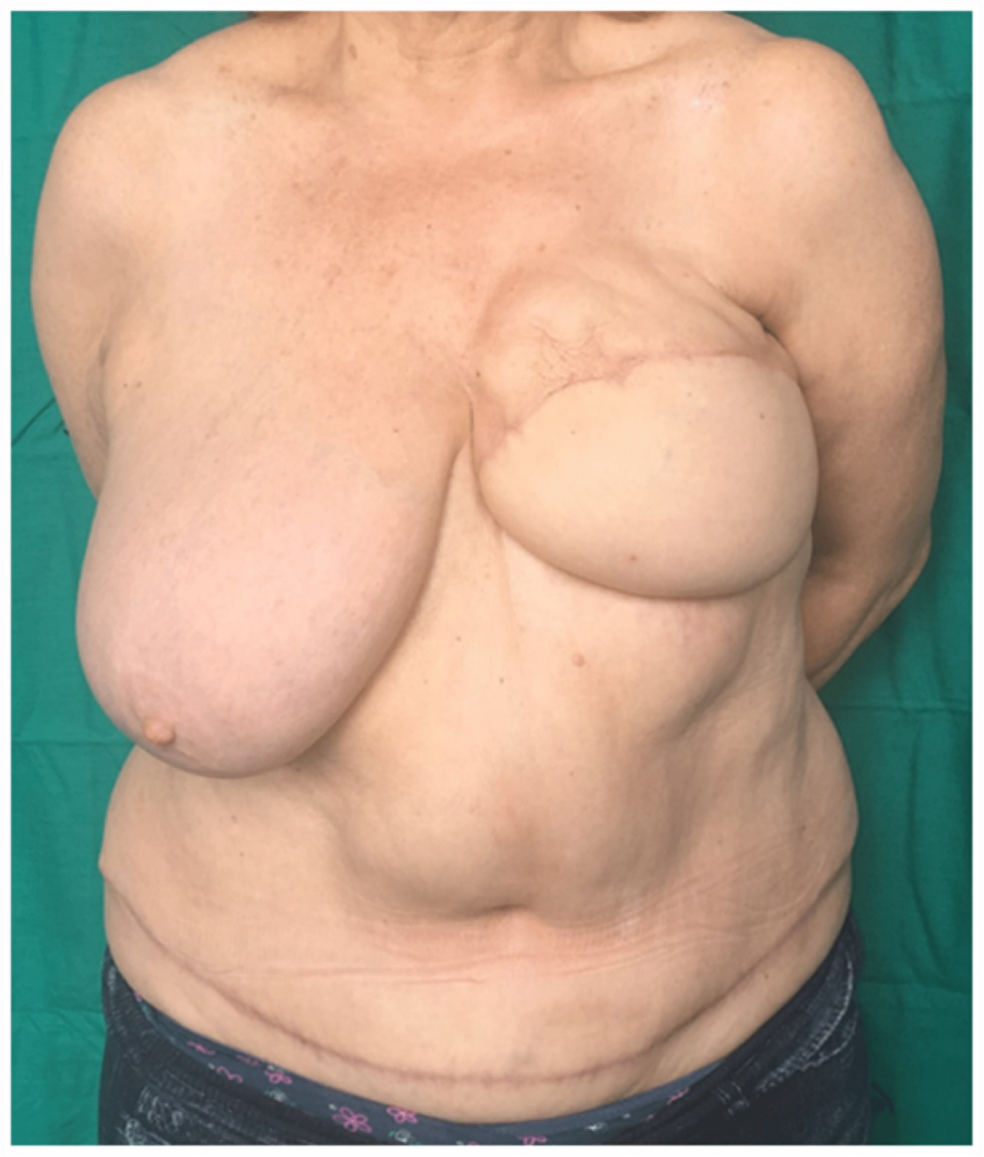
Postoperative result (case report 2).

A 66-year-old woman underwent delayed left breast reconstruction with a DIEAP flap after modified radical mastectomy, chemotherapy and radiotherapy for breast cancer. Preoperative angio-CT was performed for surgical planning and perforator selection. The largest perforator was identified from the right medial row. During dissection, the SIEVs were preserved bilaterally. At the end of the flap dissection, before the ligation of its pedicle, there was a 20-minute wait for flap assessment. Signs of venous congestion appeared and an intra-flap rerouting of the venous blood outflow was performed in situ (Figure 5). The intra-flap rerouting consisted of a reverse-flow venous anastomosis between the ipsilateral SIEV and one of the DIEV comitantes. The signs of venous congestion did not improve with this procedure, possibly due to a superficial system dominance. The pedicle was then ligated, the flap transferred to the recipient area, and the anastomoses to the recipient vessels were performed. The arterial anastomoses were performed between the DIEA and the IMA, and since there was only one recipient IMV, we dissected the DIEV comitantes previously anastomosed to the SIEV. It was ligated proximally, and the proximal stump was used for anastomosis to the IMV (Figures 6-7). Thus, the DIEV comitantes were used as a venous graft to allow tension-free communication between the SIEV and the receptor IMV. We witnessed an immediate recovery of the venous congestion signs. The usual six-day standardized anticoagulation protocol was followed. No postoperative sequelae were observed (Figure 8).

## Discussion

Intraoperative venous congestion of the DIEAP flap is the result of insufficient venous outflow drainage through the deep venous system, which is the only path for the venous outflow after the flap dissection [8]. Although angio-CT is a useful tool to explore the anterior abdominal wall anatomy and select the best perforators, it does not allow for the preoperative identification of dominant superficial system predictors or the anticipation of the venous congestion risk [4,7].

The correct selection of the perforating veins is essential for the prevention of venous congestion [8]. Schaverien et al. [16] proved that the risk of venous congestion is highly associated with the absence of nuclear magnetic resonance angiography (angio-NMR) of direct communicating veins between the perforating veins and the superficial system. Perforating veins with direct communications to the superficial system are generally larger and are more likely to be located in the medial row. According to this study, 76% of the perforators in the medial row and 57% in the lateral row exhibit direct communication with the superficial venous system. There was no correlation between the SIEV size in preoperative angio-NMR and the venous congestion rate [16]. According to Ochoa et al., there is an inverse correlation between previous surgeries of the abdominal wall and the venous congestion rate [8], probably because the previous ligation of the SIEV helps in opening the communicating and perforating veins, increasing venous drainage through the deep system [11,17].

SIEV dissection and preservation proved to be essential as a preventive measure, allowing the use of this vessel to increase venous drainage in case of venous congestion [15,18,19]. The perforating arteries with the larger venae comitantes should always be preferred, and the inclusion of multiple perforators in the same row decreases the flow resistance [14] and increases the probability of including at least one perforator with direct communication to the superficial system [16]. However, the benefit of including multiple perforators is controversial, and some authors argue that it is not preventive for venous congestion [8] or that it may be associated with an increased risk of venous congestion [5].

After completing the dissection and before the pedicle ligation, the flap should always be assessed for the in situ appearance of venous congestion signs [5]. At this point, the DIEA is the only source of arterial blood inflow, and the venous outflow is drained by the two DIEV comitantes. In case report 1, we did not wait enough time for an appropriate flap assessment in situ. We believe that the signs of venous congestion would have shown up in situ and the problem could have been solved before the flap transfer to the recipient area. Currently, we wait 20 minutes before the pedicle ligation, as we did in case report 2.

When the perforating veins size is reduced (<1 mm) or the SIEV size is increased (>1.5 mm), signs of venous congestion may appear, due to an inability of venous drainage through the deep system [6,14,15]. In those cases, an intra-flap venous rerouting technique should be attempted in situ, before the pedicle ligation [5]. There are several techniques described in the literature, and the most widely used vein of the superficial system is the ipsilateral SIEV [5,7,8,10,18,20]. If none of the SIEVs are adequate, the superficial circumflex iliac veins (SCIV) can be used. In case of disrupted communication between the superficial and deep systems or an inappropriate perforator selection, those intra-flap rerouting techniques are effective and allow the resolution of venous congestion before the pedicle ligation [5]. In our institution, the venous flow rerouting from the superficial system to the deep system is made by performing a reverse-flow venous anastomosis between the ipsilateral SIEV and the distal stump of one of the DIEV comitantes (Figure 3).

If the intra-flap rerouting is not effective, there may be a dominant superficial system with implicit high-resistance DIEV comitantes, impaired communication between the DIEV comitantes, or an injury to the DIEV comitantes during the flap dissection. All of those causes, ultimately, lead to an impaired drainage of the venous outflow despite effective communication between the superficial and deep systems [3,5,11]. In these cases, an extra-flap rerouting through an anastomosis between the superficial system and a recipient vein should be attempted [5]. There are several techniques described in the literature with several receptor vessels available for extra-flap rerouting [4,5,7,8,10,18,20]. These techniques have to be done in the recipient area, after the pedicle ligation. In our institution, when we performed the extra-flap rerouting, we had already attempted the in situ intra-flap rerouting by anastomosing the ipsilateral SIEV to one of the DIEV comitantes. Therefore, we used the DIEV comitantes as a venous graft to link the SIEV to the IMV (Figure 7).

The patients who do not show signs of venous congestion in situ (venous drainage by the two DIEV comitantes) but develop signs after the anastomoses to the recipient vessels (drainage by one DIEV comitantes) can benefit from a second venous anastomosis between the second DIEV comitantes and a recipient vein [8].

Some studies have shown no flap loss when an extra venous anastomosis is performed to increase the venous outflow drainage [5,8]. Although there is no clear evidence in favor of the systematic venous drainage increase, Lee et al. [3] demonstrated that a prophylactic increase of venous drainage through an additional venous anastomosis reduces the risk of congestion while having little influence on flap survival [3].

## Conclusions

Scrupulous preoperative planning is very important as the selection of large perforators increases the likelihood of direct venous connections with the superficial system and reduces the risk of venous congestion. The dissection and preservation of the SIEV proved to have a fundamental role as a prophylactic measure, as it can be used for rescue. The current approach of our institution is assessing all the DIEAP flaps in situ, and if venous congestion signs are evident, we systematically perform an intra-flap rerouting before the ligation of the pedicle. This maneuver can be sufficient to solve the problem. However, when it is not effective, we proceed to an extra-flap rerouting, utilizing the intra-flap anastomosis previously performed as part of the extra-flap rerouting.
